# Dietary intake of flavonoid subclasses and risk of colorectal cancer: evidence from population studies

**DOI:** 10.18632/oncotarget.8562

**Published:** 2016-04-02

**Authors:** Xingkang He, Lei-min Sun

**Affiliations:** ^1^ Department of Gastroenterology, Sir Run Run Shaw Hospital, Zhejiang University Medical School, Hangzhou 310016, China; ^2^ Institute of Gastroenterology, Zhejiang University (IGZJU), Hangzhou 310016, China

**Keywords:** flavonoids, isoflavones, procyanidins, colorectal cancer

## Abstract

**Objective:**

To systematically evaluate the relationship between flavonoids intake and colorectal cancer risk by conducting a meta-analysis.

**Results:**

Our meta-analysis included 18 studies involving 16,917 colorectal cancer cases in 559,486 participants in relations to flavonoids intake during six to twenty-six years of follow-up. Our results indicated that specific flavonoid subclasses, such as procyanidins (OR = 0.75; 95% CI, 0.66–0.86) and isoflavones (OR = 0.87; 95% CI, 0.78–0.98), showed protective effects against colorectal cancer risk. There was no enough evidence indicating that increased consumption of total flavonoids were significantly associated with reduced risk of colorectal cancer (OR = 0.94, 95% CI, 0.81–1.09). There was no publication bias across studies.

**Methods:**

We performed a systematic search of PubMed, Web of Science and the Cochrane Library databases for relevant articles before December 2015. A random-effects model was used to estimate summary odds ratios and 95% confidence intervals (CIs) for associations between flavonoids and colorectal cancer risk. We assessed heterogeneity among studies by the Cochran Q and I^2^ statistics.

**Conclusions:**

Our meta-analysis provides comprehensive evidence and partly supported the hypothesis that higher habitual intake of foods rich in procyanidins and isoflavones may potentially decrease colorectal cancer incidence. More prospective studies are warranted to verify this protective association.

## INTRODUCTION

Colorectal cancer is the third most prevalent cancer and the third leading cause of cancer-related death in men and women in America [1]. In 2014, there were estimated to be 136,830 new colorectal cancer patients and 50,310 cancer-related deaths in the United States [2]. Over the past decades, there has been substantial progress in reducing colorectal cancer morbidity and mortality due to screening programs and advanced therapies [3]. Because of huge economic burden of this disease, there is still an urgent need to tailor colorectal cancer prevention strategies.

Current epidemiologic studies to date have suggested that dietary factors play a crucial role in the development of colon cancer [4, 5], and high fruit and vegetable was generally implicated in the prevention of colorectal cancer [6–8]. A possible protective role of flavonoids against colorectal cancer has been of enormous interest recently [9–13]. Flavonoids are a diverse group of polyphenolic compounds widely available in plant-based foods, such as fruits, vegetables, herbs, tea, and juices [14]. According to their chemical structure, flavonoids can be classified as flavones, flavonols, flavanones, flavanols (flavan-3-ols), anthocyanins, isoflavoness [15]. Besides, proanthocyanidins are another important subclass of polyphenols [16].

In recent decades, accumulating studies have been conducted to investigate the relationship between diet flavonoids and colorectal cancer incidence. However, existing data is still conflicting. For example, several studies indicated that flavonoids were inversely associated with colon cancer risk [12, 13, 17–21], however, other prospective cohort studies generally failed to detect such relationship [9, 10, 22, 23]. In addition, a randomized controlled trial showed that higher intake of flavonols was associated with a 76% reduced recurrence of advanced adenoma [24]. Because of differences in study design and type of flavonoids, various studies yielded inconsistent results.

Nevertheless, Experimental studies provide evidence for potential mechanisms that relate flavonoids to cancer risk. For example, flavonoids could inhibit growth of colon cancer cell lines and colorectal carcinogenesis in animal models [25, 26]. Different subclasses of flavonoids may have varying capacities to suppress neoplasm. There are several anti-carcinogenic mechanisms of flavonoids, including antioxidative and anti-inflammatory activities [27], induction of apoptosis and suppression of angiogenesis [28, 29].

To better understand this association, we performed a meta-analysis of available studies to comprehensively evaluate dietary flavonoids intake as well as flavonoid specific subclasses in relation to colorectal cancer risk.

## RESULTS

**Figure 1:**
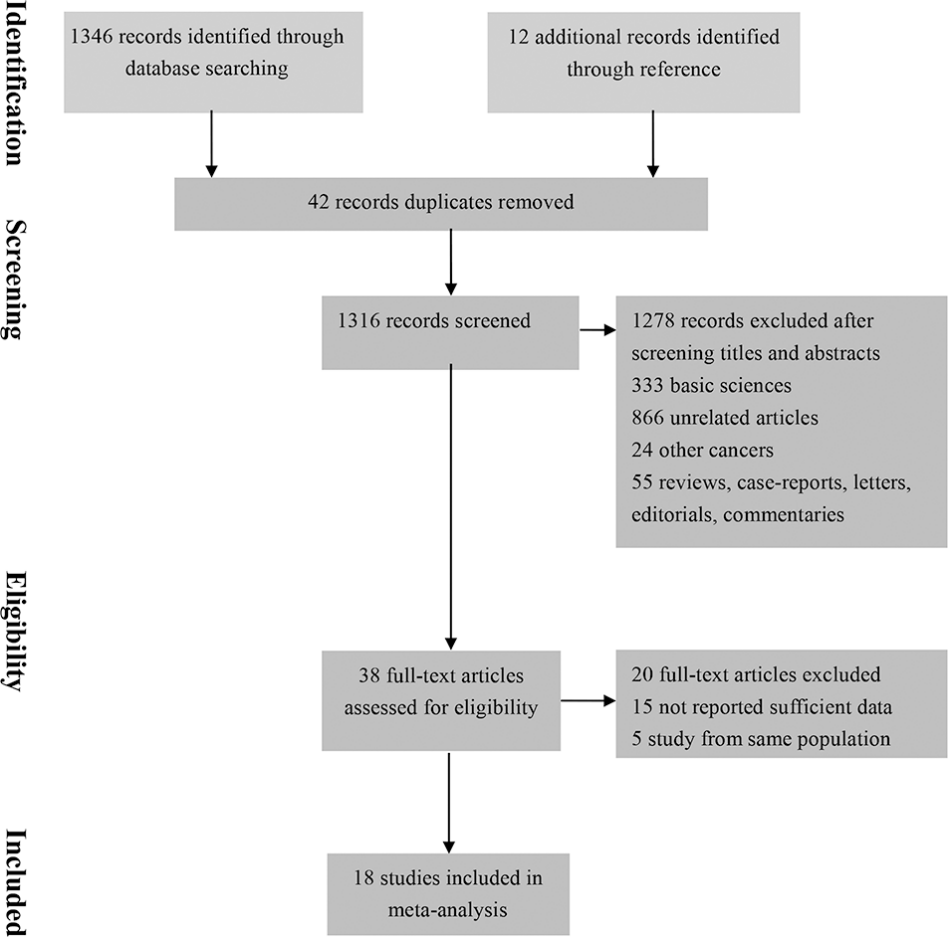
Flow diagram summarizing study identification and selection

Total 1,358 studies were identified through the literature searches. After review of the titles and abstracts, 1,278 studies were excluded and remaining 38 studies were reviewed with the full texts. Thus 18 studies were finally included in final meta-analysis. (Figure 1)

### Characteristics and quality of included studies

The characteristics of selected studies were outlined in Table 1. We identified five studies on total flavonoids intake and CRC risk and 16 studies that assessed subclasses of flavonoid consumption in relation to CRC incidence. These studies involved 559,486 participants with 16,917 CRC cases. Nine of them were prospective cohort studies and remaining were case-control studies. These studies were conducted in Europe (*n* = 9), Asia (*n* = 6), and America (*n* = 3). Food frequency questionnaires (FFQs) were used to assess exposure to certain dietary flavonoids in all but three studies [10, 18, 30], which adopted interview, food records, and diet diaries. The diagnosis of colorectal cancer was based on histologic findings or data from cancer registry.

**Table 1A:** Characteristics of included case-control studies on dietary flavonoids and risk of colorectal cancer

Study	Design	Location/ Setting	Exposure Ascertainment	Outcome assessment	Total subjects	Colon cancer cases	Confounding variables adjusted
Shin et al. 2015	Case-control hospital-based	Korea	Validated FFQ	Medical record	3570	901	1,4,5,7
Zamora-Ros et al. 2013	Case-control hospital-based	Spanish	Validated FFQ	Histological confirmed	825	424	1,2,4,5,6,7,8,10,12,15
Budhathoki et al. 2011	Case-control population-based	Japan	Computer-assisted interview	Histological confirmed	1631	816	1,2,3,4,5,13,14,15,16
Rossi et al. 2010	Case-control hospital-based	Italy	Validated FFQ	Histological confirmed	6107	1953	1,2,3,4,5,10,11,13,15
Ward et al. 2010	Prospective case-control	Norfolk	Diet diaries	Cancer Registry	1103	220	1,3,5,6,7,8,10,12,15,16
Kyle et al. 2009	Case-control population-based	Britain	Validated FFQ	Histological confirmed	672	264	1,10,12,15,16
Theodoratou et al. 2007	Case-control population-based	Britain	Validated FFQ	Histological confirmed	2912	1456	3,4,5,6,7,9,10,12,15
Cotterchio et al. 2006	Case-control population-based	America	FFQ	Histological confirmed	2985	1095	1,2,10
Rossi et al. 2006	Case-control hospital-based	Italy	Validated FFQ	Histological confirmed	6107	1953	1,2,3,5,6,10,13,15

Abbreviation: FFQ, food frequency questionnaire

1 = age, 2 = sex, 3 = body mass index, 4 = alcohol, 5 = physical activity, 6 = smoke, 7 = fibre, 8 = meat intake, 9 = fruit/vegetable intake, 10 = total energy intake (kcal/day), 11 = education 12 = NSAID, 13 = study location, 14 = occupation, 15 = family history of colorectal cancer, 16 = dietary supplements (calcium, *n*-3 polyunsaturated fatty acids, manganum, riboflavin, vitamin C, vitamin E, folate).

**Table 1B:** Characteristics of included cohort studies on dietary flavonoids and risk of colorectal cancer

Study	Design	Location	Time period; (years)	Exposure Ascertainment	Outcome assessment	Total subjects	Colon cancer cases	Confounding variables adjusted
Nimptsch et al. 2015	Cohort	America	26	Validated FFQ	Histological confirmed	118842	2519	1,3,4,5,6,8,12,15, 16,17
Simons et al. 2009	Cohort	Netherlands	13.3	Validated FFQ	Cancer Registry	120852	2485	1,3,4,5,6,8,15
Yang et al. 2009	Cohort	China	6.4	Validated FFQ	Medical record	68412	321	1,3,5,8,9,11,15,16, 19,20,21
Wang et al. 2009	Cohort	America	11.5	Validated FFQ	Medical record	38408	305	3,4,5,6,7,9,15,16, 20,21
Butler et al. 2008	Cohort	Singapore	10	Validated FFQ	Cancer registry	61321	961	1,2,18,6,4,3,11, 5,15,10
Akhter et al. 2008	Cohort	Japan	7.6	Validated FFQ	Medical record	83063	886	1,3, 4,5, 6,8,9,13, 16,18, 21
Mursu et al. 2008	Cohort	Finnish	16.2	Food records	Cancer registry	2590	55	1,3,6,5,4,3,10,16,7
Oba et al. 2007	Cohort	Japan	8	FFQ	Histological confirmed	30221	213	1,3,4,5,6,16,21
Knekt et al. 2002	Cohort	Finnish	6	FFQ	Cancer Registry	9865	90	1,2,3,6,13,14

Abbreviation: FFQ, food frequency questionnaire

1 = age, 2 = sex, 3 = body mass index, 4 = alcohol, 5 = physical activity, 6 = smoke, 7 = fibre, 8 = meat intake, 9 = fruit/vegetable intake, 10 = total energy intake (kcal/day), 11 = education 12 = NSAID, 13 = study location, 14 = occupation, 15 = family history of colorectal cancer, 16 = dietary supplements (calcium, *n*-3 polyunsaturated fatty acids, manganum, riboflavin, vitamin C, vitamin E, folate), 17 = history of endoscopy, 18 = history of diabetes mellitus, 19 = household income, 20 = menopausal status, 21 = current use of female hormones.

The overall methodological quality of studies was summarized in Table 2. Using the Newcastle–Ottawa scale (NOS) quality tool, the score of all the studies ranged from 6 to 9, indicating moderate to high quality.

**Table 2A:** Newcastle-Ottawa scale for assessment of quality of in included Cohort studies

Author	Quality assessment criteria								Overall QualityScore(max = 9)
	Selection				Comparability	Outcome			
	Representativeness of exposed cohort?	Selection of the non-exposed cohort?	Ascertainment of exposure?	Outcome of interest was not present at start of study?	Study control forage/gender and additional factor?	Assessment of outcome?	Was follow-up long enough for outcome to occur?	Adequacy of follow-up of cohorts?	
Nimptsch et al. 2015	*	*	*	*	**	*	*	*	9
Simons et al. 2009	-	*	*	*	**	*	*	*	8
Yang et al. 2009	-	*	*	*	**	*	*	-	7
Wang et al. 2009	-	*	*	*	**	*	*	-	7
Akhter et al. 2008	*	*	*	*	*	*	*	*	8
Mursu et al. 2008	-	*	*	*	*	*	*	*	7
Butler et al. 2008	*	*	*	*	**	*	*	*	9
Oba et al. 2007	*	*	-	*	*	*	*	*	7
Knekt et al. 2002	*	*	-	*	*	*	*	-	6

Each asterisk represents if individual criterion within the subsection were fulfilled.

**Table 2B:** Newcastle-ottawa scale for assessment of quality of in included case-control studies

Author	Quality assessment criteria								Overall Quality
	Selection				Comparability	Outcome			
	Is the case definition adequate?	Representativeness of cases?	Selection of control?	Definition of control?	Study control for age/gender and additional factor?	Ascertainment of exposure?	Same method of cases/controls?	Non-response rate	
Shin et al. 2015	*	*	-	*	*	*	*	-	6
Zamora-Ros 2013	*	*	-	*	**	*	*	-	7
Budhathoki et al. 2011	*	*	*	*	**	*	*	-	8
Rossi et al. 2010	*	*		*	**	*	*	*	8
Ward 2010	*	-	-	*	**	*	*	-	7
Kyle et al. 2009	*	*	*	*	**	*	*	*	9
Theodoratou et al. 2007	-	*	*	*	**	*	*	*	8
Cotterchio et al. 2006	*	*	-	*	*	*	*	*	7
Rossi et al. 2006	*	*	*	-	*	*	*	-	6

Each asterisk represents if individual criterion within the subsection were fulfilled.

### Total diet flavonoids intake and colorectal cancer risk

Five studies investigated the association of total flavonoids with incidence of colorectal cancer. The combined results indicated that no statically significant difference in colorectal cancer risk between the highest flavonoid intake and the lowest (OR = 0.94, 95% CI 0.81–1.09) (Supplementary Figure S1). There was no evidence of significant heterogeneity (*P* = 0.44, *I*^2^ = 0.0%). Furthermore, results for both case-control (OR = 0.81; 95% CI 0.50–1.29) and cohort studies (OR = 1.00; 95% CI 0.74–1.35) were similar. The Egger's test (*P* = 0.59) and Begg's test (*P* = 0.62) showed no evidence of publication bias in this meta-analysis.

### Subclasses of diet flavonoids consumption and colorectal cancer incidence

#### Flavones

The correlation between high vs low intake of flavones and CRC risk were presented in five studies. The summary analysis yielded a combined risk estimate of 0.91 (95% CI, 0.78–1.05) with some evidence of heterogeneity (*I*^2^ = 56.9%, *P* = 0.04) (Supplementary Figure S2). There was no publication bias in analysis. We further conducted subgroup analyses by study design, sex and tumour location (Table 3). A significant association was found only for flavones intake and rectal cancer risk. However, no reduced risk of colorectal cancer was observed in the subgroup analyses by sex and design.

**Table 3:** Stratified analyses of flavonoid subclasses and colorectal cancer risk

Subgroup analysis		Pooled OR	95% CI	Heterogeneity I^2^ (%)	*P* Value
**Flavones**					
**Design**	Case-control	0.83	(0.69,1.14)	75.6%	0.017
	Cohort	0.98	(0.88,1.08)	0	0598
**Gender**	Male	0.95	(0.87,1.05)	6.9%	0.342
	Female	0.95	(0.87,1.04)	0	0.564
**Site of tumour**	Colon	0.88	(0.68,1.13)	73.1%	0.011
	Rectum	0.82	(0.70,0.97)	0	0.608
**Flavonols**					
**Design**	Case-control	0.70	(0.62,080)	0	0537
	Cohort	1.01	(0.91,1.23)	4.9%	0.349
**Gender**	Male	0.88	(0.77,1.01)	39.7%	0.191
	Female	0.87	(0.71,1.05)	80.4%	0.006
**Site of tumour**	Colon	0.784	(0.62,1.00)	67.1%	0.016
	Rectum	0.82	(0.63,1.08)	50.5%	0.089
**Flavanones**					
**Design**	Case-control	1.14	(0.93,1.38)	42.5%	0.156
	Cohort	0.96	(0.84,1.10)	0	0.888
**Gender**	Male	1.00	(0.90,1.11)	0	0.581
	Female	0.98	(0.89,1.08)	0	0.716
**Site of tumour**	Colon	1.03	(0.92,1.15)	0	0.653
	Rectum	0.94	(0.80,1.11)	0	0.825
**Flavanols**					
**Design**	Case-control	0.80	(0.64,0.99)	51.8%	0.101
	Cohort	1.01	(0.86,1.18)	41.3%	0.182
**Gender**	Male	1.06	(0.94,1.19)	36.3%	0.208
	Female	0.96	(0.86,1.07)	39.2%	0.193
**Siteof tumour**	Colon	0.88	(0.69,1.12)	68.3%	0.013
	Rectum	0.87	(0.74,1.02)	0	0.542
**Anthocyanins**					
**Design**	Case-control	0.68	(0.56,0.83)	0	0.667
	Cohort	0.92	(0.67,1.28)	17%	0.272
**Gender**	Female	0.87	(0.66,1.13)	78.6%	0.009
	Male	0.89	(0.82,0.96)	0	0.862
**Site of tumour**	Colon	0.79	(0.61,1.02)	55.3%	0.107
	Rectum	0.88	(0.67,1.00)	72.3%	0.027
**Isoflavones**					
**Design**	Case-control	0.85	(0.72,1.01)	71.3%	0.002
	Cohort	0.93	(0.83,1.04)	0	0.518
**Gender**	Male	0.920	(0.78,1.08)	50.5%	0.049
	Female	0.940	(0.84,1.06)	0	0.469
**Site of tumour**	Colon	0.86	(0.73,1.00)	35.4%	0.158
	Rectum	0.93	(0.78,1.10)	25.1%	0.237
**Procyanidins**					
**Design**	Case-control	0.75	(0.66,0.86)	0	0.633
	Cohort	-*	-	-	-
**Gender**	Male	0.88	(0.80,0.98)	0	0.655
	Female	0.84	(0.74,0.96)	0	0.695
**Site of tumour**	Colon	0.81	(0.69,0.96)	0	0.555
	Rectum	0.66	(0.54,0.80)	0	0.522

* no cohort studies were included in analysis for procyanidins.

#### Flavonols

The correlation between high vs low intake of flavonols and CRC risk were presented in six studies. The summary risk estimate was 0.86 (95% CI, 0.71–1.03), with considerable heterogeneity (*I*^2^ = 73.9%, *P* = 0.001) (Supplementary Figure S3). There was no publication bias in analysis. In subgroup analyses, the reduced risk of colorectal cancer was observed in pooled estimates of case-control studies, but not for cohort studies. There was no other significant association detected.

#### Flavanones

The correlation between high vs low intake of flavanones and CRC risk were presented in six studies. The summary risk estimate was 1.05 (95% CI, 0.92–1.19), with no evidence of heterogeneity (*I*^2^ = 27%, *P* = 0.23) (Supplementary Figure S4). No publication bias was detected in the analysis. There were no significant associations found in subgroup analyses.

#### Flavanols

The correlation between high vs low intake of flavanols and CRC risk were presented in seven studies. The summary risk estimate was 0.90 (95% CI, 0.78–1.04), with some heterogeneity (*I*^2^ = 56.8%, *P* = 0.03) (Supplementary Figure S5). No publication bias was detected in the analysis. In subgroup analyses, the association was significant in case-control studies, but not in cohort studies.

#### Anthocyanins

The correlation between high vs low intake of anthocyanins and CRC risk were presented in four studies. The summary risk estimate was 0.78 (95% CI, 0.61–1.01), with considerable heterogeneity (*I*^2^ = 60.2%, *P* = 0.057) (Supplementary Figure S6). There was no publication bias in the analysis. The subgroup analysis by design produced a significant summary risk estimate for case-control studies, but not for cohort. Furthermore, reduced risk of CRC was observed in male, but not for female.

#### Isoflavones

The correlation between high vs low intake of isoflavones and CRC risk were presented in eleven studies. The summary risk estimate was 0.87 (95% CI, 0.78–0.98), with considerable evidence of heterogeneity (*I*^2^ = 59.5%, *P* = 0.006) (Supplementary Figure S7). The result should be interpreted with caution, since significant heterogeneity existed among included studies. No publication bias was detected in the analysis. We conducted stratified analyses of eleven studies between isoflavones and colorectal cancer risk to determine the impact of differences in study design, gender, and site of tumour. No significant association was detected in either analyses.

#### Procyanidins

The correlation between high vs low intake of procyanidins and CRC risk were presented in four studies. The summary risk estimate was 0.75 (95% CI, 0.66–0.86), with no evidence of heterogeneity (*I*^2^ = 0, *P* = 0.63) (Supplementary Figure S8). There was no evidence of publication bias in analysis. The reduced risk of CRC was not only observed in male and female, but also in colon and rectum. Since studies included in this meta-analysis were all case-control, this protective association should be interpreted with caution.

## DISCUSSION

In the present study, five epidemiologic studies that assessed the association between total flavonoids consumption and colorectal cancer risk in humans. Other studies evaluated the relationship between several subclasses of flavonoid and CRC risk. To our knowledge, this is the most comprehensive meta-analysis and evidence from our study indicated that total flavonoids intake were not significantly associated with reduced CRC risk. The lack of association is likely explained by the fact that limited numbers of included studies, which leaded limited power to detect an association. Furthermore, we assess potential relationships between flavonoid subclasses and CRC risk, respectively. Isoflavones and procyanidins, but not other subclasses, were inversely associated with the reduced CRC incidence. Thus, these findings partially supported flavonoid subclasses might be considered as promising candidates for potential chemopreventive agents, such as aspirin, metformin, vitamin D [31–35].

Flavonoids, as a diverse group of polyphenol, are considered as a potential anti-carcinogenic agent. Although our analyses provided some evidence of an inverse association between specific subclasses and CRC incidence, several experimental studies, both *in vitro* and *in vivo*, supported its protective role against CRC. Flavonoids have varying capacities to inhibit the development of colorectal cancer, for example, acting as antioxidants [27, 36, 37], anti-inflammatory agents [27, 38], anti-proliferative agents [39]. *In vitro*, flavonoids inhibiting growth of cancer cells through suppression of p21-RAS and DNMT expression [40, 41]. In addition, flavones induced effectively apoptosis through down-regulation of cyclooxygenase-2 (COX-2), nuclear transcription factor kappaB [42, 43]. However, effects of flavonoids among humans cannot be easily extrapolated from basic research. Since concentrations of flavonoids used in experimental studies were hardly reached through dietary intake, the evidence is less conclusive [39]. Therefore, whether flavonoids intake protect against colorectal cancer still needs further confirmation from epidemiologic studies and randomized clinical trials.

Our meta-analyses showed that higher consumption of isoflavones and procyanidins might be associated with lower risk of colorectal cancer. Previous meta-analysis [44] presented that soy food intake was associated with a 21% reduction in colorectal cancer risk among high intake groups in women. Isoflavones, a bioactive component rich in soy food, might have potential capacity in inhibition of cancer [45]. Our combined analysis also partially supported this hypothesise. Isoflavones, also known as phytoestrogens, might exerted anti-carcinogenic effects through hormonal and non-hormonal pathways [46, 47]. Several epidemiological studies had reported a reduced risk for CRC among high isoflavones intake [11, 12, 18, 21, 48]. The protective association was more prominent among post-menopausal women than pre-menopausal women. However, our stratified analyses were unable to detect this significant association and this may relate to limited number of included studies. Procyanidins, also known as condensed tannins, occur ubiquitously in plants. They can exert a wide variety of beneficial biological effects, such as antioxidant anti-inflammatory and anti-cancer [49]. Furthermore, this protective association was still consistent among subgroup analyses.

It is important to note several limitations of our analysis. Firstly, most results included in our analyses were case-control studies. Although the methodological quality of these observational studies was medium to high, case-control studies were prone to introduce recall bias. More prospective cohort studies need to test this association. Secondly, it was a challenge to evaluate the quantity of flavonoids intake accurately. Since the FFQ included limited flavonoid-rich food items and intake ranges, the specifically designed FFQ for flavonoids intake should be developed. In addition, flavonoid contents in food may vary depending on other factors, such as species, season and ripeness. These factors may introduce additional measurement error and therefore misestimate the relationship between flavonoids intake and cancer risk. Thirdly, bioactive compounds in food are complex and highly correlated. It is hard to completely tease apart their interaction and rule out the possibility that potential unknown components in food may co-associate with flavonoids. Further intervention studies may be required to elucidate whether the main protective effects are actually due to these flavonoids.

In conclusion, our analyses supported that several subclasses of flavonoid, procyanidins and isoflavones, may potentially protect against colorectal cancer. our results are still promising despite of the lack of sufficient evidence to show that total flavonoids were associated with reduced risk of colorectal cancer in this meta-analysis. Well-designed cohort studies are needed to further investigate the effects of exposure to dietary flavonoids and subclasses.

## MATERIALS AND METHODS

### Search strategy

We (HXK and SLM.) conducted a systemic search of PubMed, Web of Science and the Cochrane Library databases for all relevant studies before December 2015 independently. The following text and/or medical subject heading terms were used in the literature search: (1) flavonoid*, flavone*, flavonol*, flavanone*, flavanol*, anthocyanin*, isoflavone*, procyanidin*, (2) neoplasm, cancer, tumour, (3) colorectal, colon, rectal, large bowel. In addition, we scanned and examined the reference lists in relevant articles manually.

### Eligibility criteria

Studies were eligible for this meta-analysis if they met the following criteria: (1) original article; (2) case-control or cohort studies; (3) evaluating the association between flavonoids or subclasses intake and CRC risk, and (4) reporting adjusted risk estimates with 95% CIs. In addition, case reports, editorials, reviews, animal studies or *in-vitro* researches were excluded. Besides, studies lacking relevant data also were excluded. When data from several publications were overlapping, we selected the publication with the most comprehensive data for inclusion in the meta-analysis.

### Data extraction and quality assessment

Two authors (HXK and SLM.) independently reviewed titles and abstracts of potentially eligible articles identified by the search strategy. Two researchers (HXK and SLM.) independently extracted the following information from included studies: the first author's name, year, location, duration of follow-up, total subjects, colorectal cancer cases and adjustments for confounders. From these studies, we extracted the risk estimate of the highest relative to the lowest intake of dietary flavonoids and subclasses. Two authors independently evaluated the quality of included studies using the Newcastle–Ottawa Scale [50]. Any disagreements were resolved by discussions.

### Statistical analysis

We calculated summary odds ratios with 95% CI using a random-effects model [51], since considering between-study variation. Adjusted risk estimates reported in studies were used for meta-analysis in order to account for confounding factors. We assessed heterogeneity between by Cochran's *Q* test and I^2^ statistics [52]. Significant heterogeneity was indicated if *P* value was < 0.1 or I^2^ value greater than 50% [53]. Prespecified subgroup analyses were performed to assess the potential modifying effects of the following variables on outcomes: study design, gender and tumour location. Besides we performed sensitivity analyses to test the robustness of our combined effects. We used the Egger's and Begg's test to assess publication bias [53]. A *P* value < 0.05 (except for Cochran's *Q* test) was considered statistically significant and all *P* values were two tailed. All statistical analyses were conducted using Stata software (version 13.0; Stata Corp, College Station, TX, United States).

## SUPPLEMENTARY MATERIALS FIGURES


