# Supplementary material for: Dietary intake of flavonoid subclasses and risk of colorectal cancer: evidence from population studies
Source: Oncotarget. 2016 Apr 2;7(18):26617–27. doi: 10.18632/oncotarget.8562 (PMC5042003; doi:10.18632/oncotarget.8562)
Supplement: Supplementary file 1 [file oncotarget-07-26617-s001.pdf]

Dietary intake of flavonoid subclasses and risk of colorectal cancer: evidence from population studies

Supplementary Materials

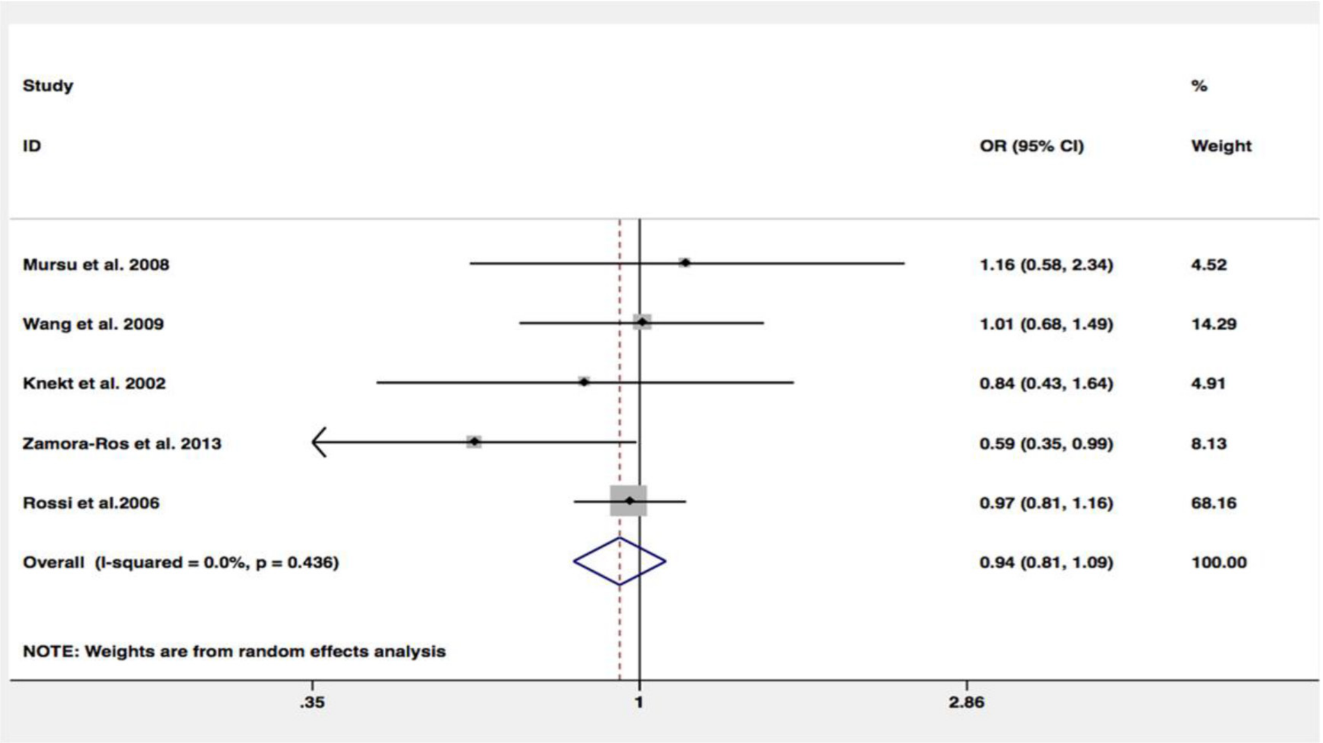

Supplementary Figure S1: Forest plot of total flavonoids intake and colorectal cancer risk.

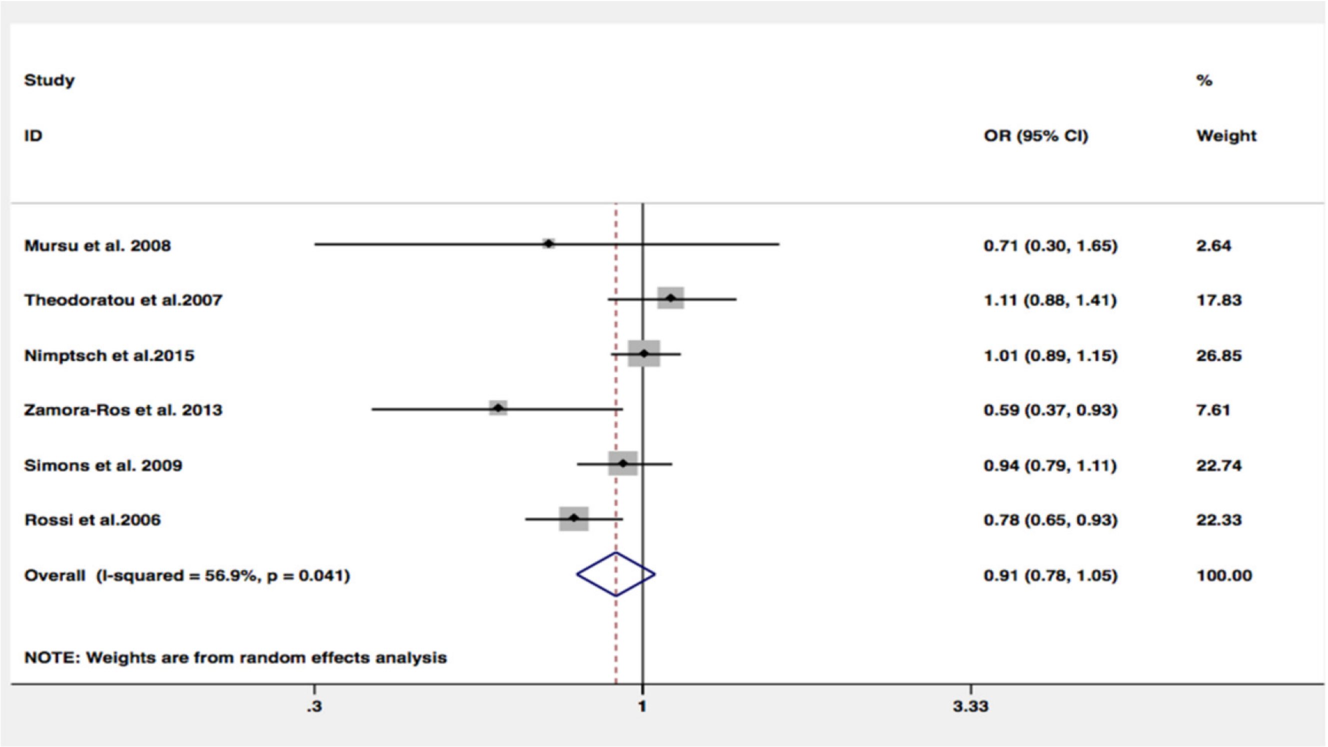

Supplementary Figure S2: Forest plot of flavones intake and colorectal cancer risk.

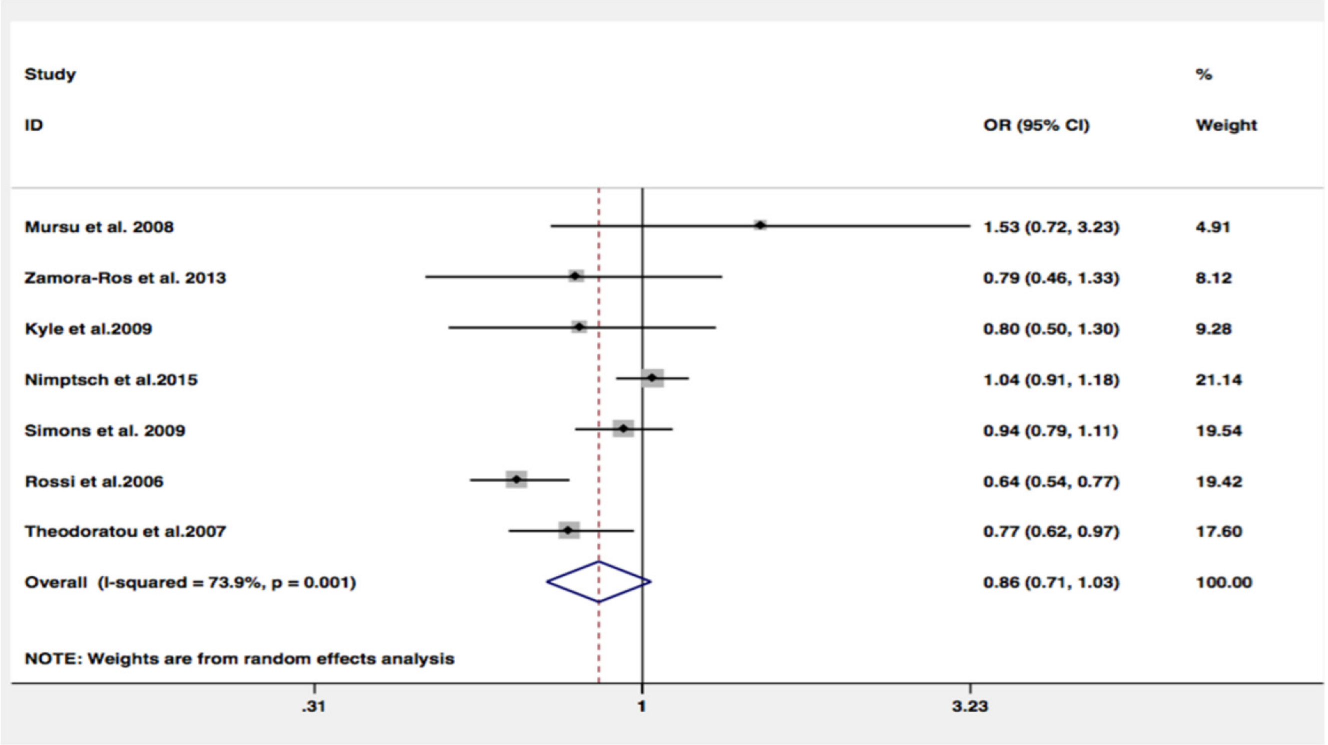

Supplementary Figure S3: Forest plot of flavonols intake and colorectal cancer risk.

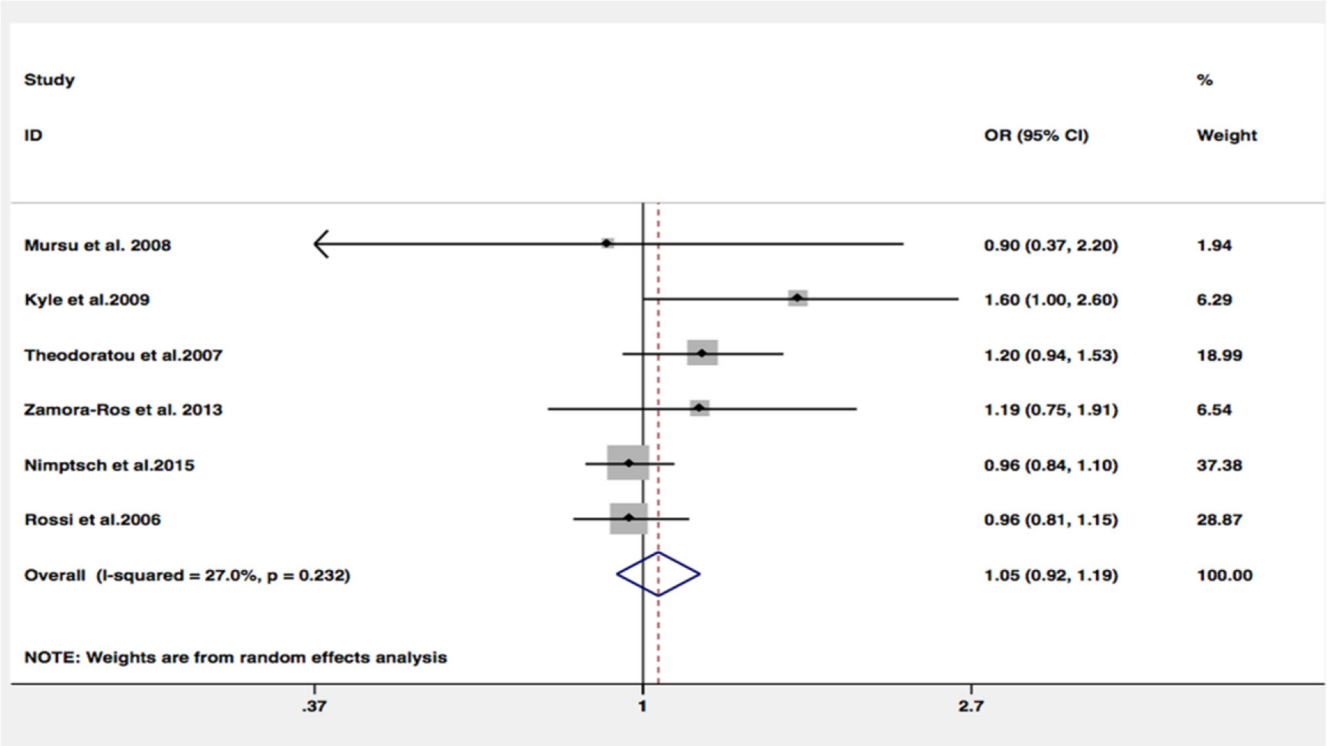

Supplementary Figure S4: Forest plot of flavanones intake and colorectal cancer risk.

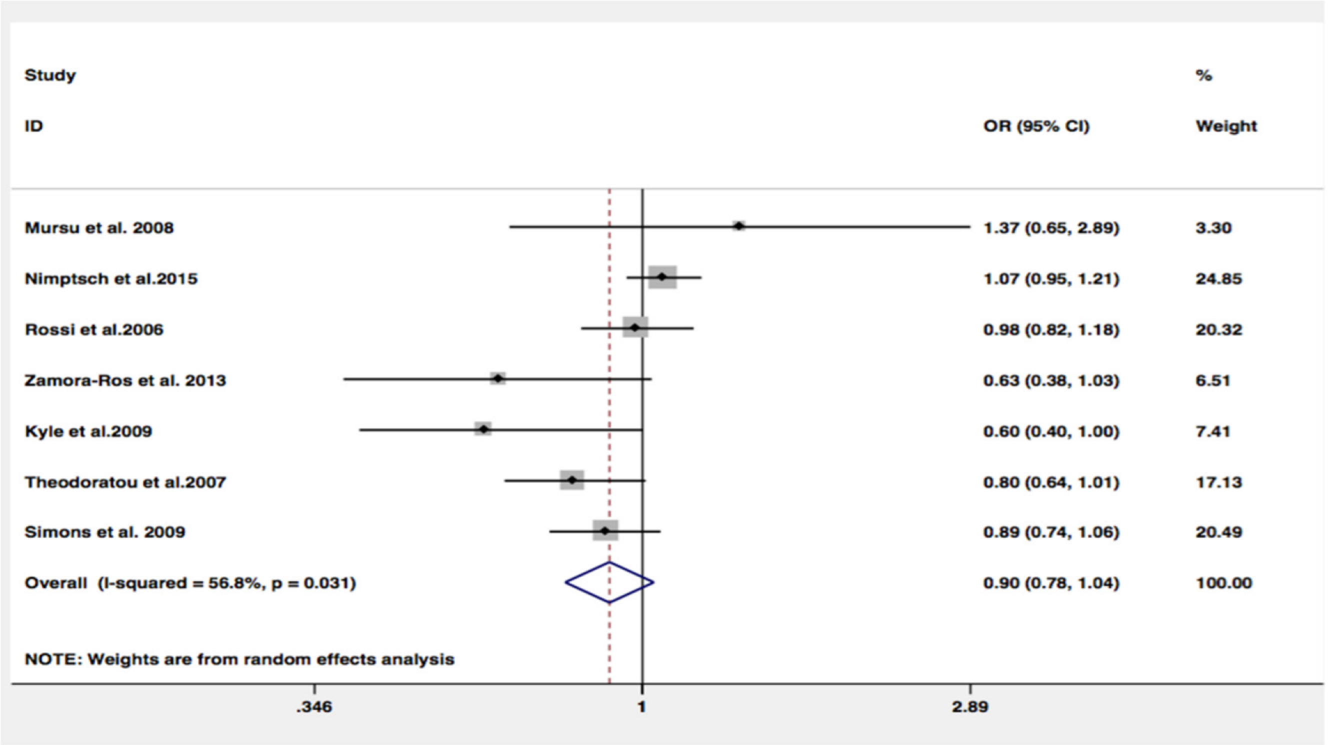

Supplementary Figure S5: Forest plot of flavanols intake and colorectal cancer risk.

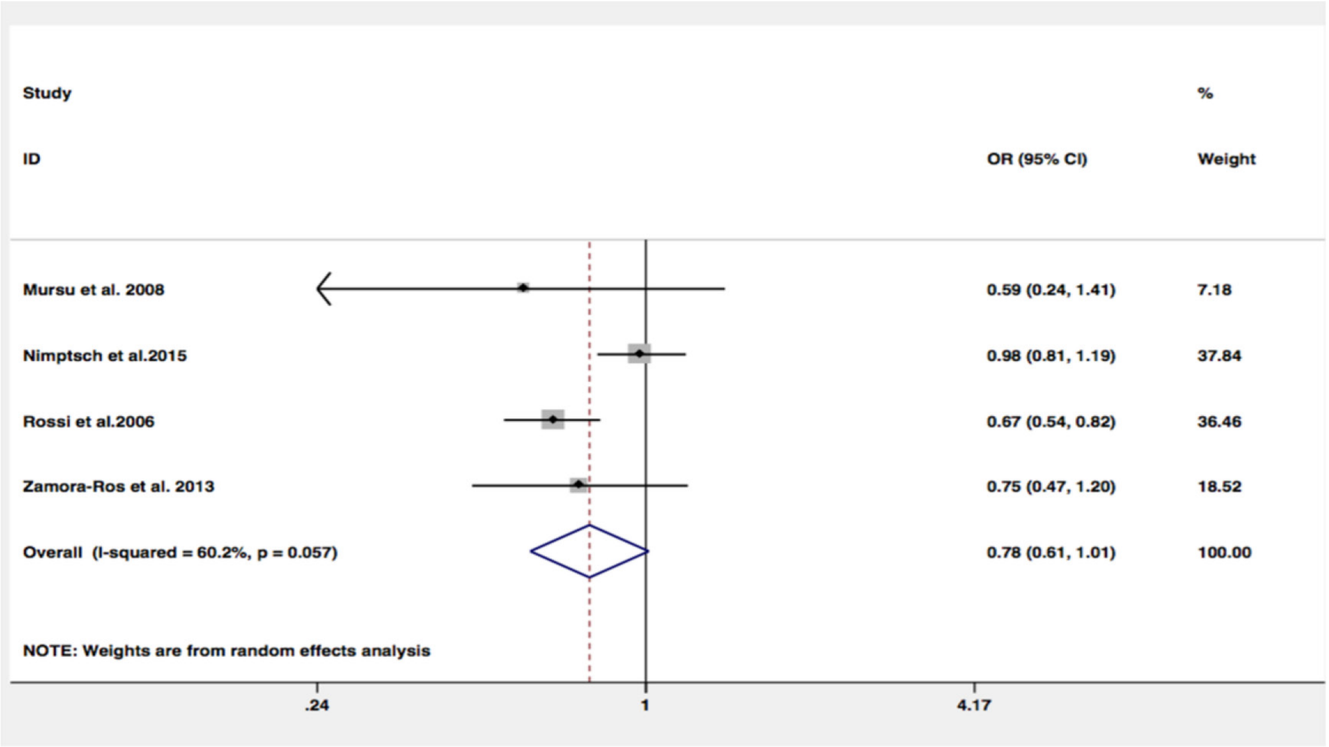

Supplementary Figure S6: Forest plot of anthocyanins intake and colorectal cancer risk.

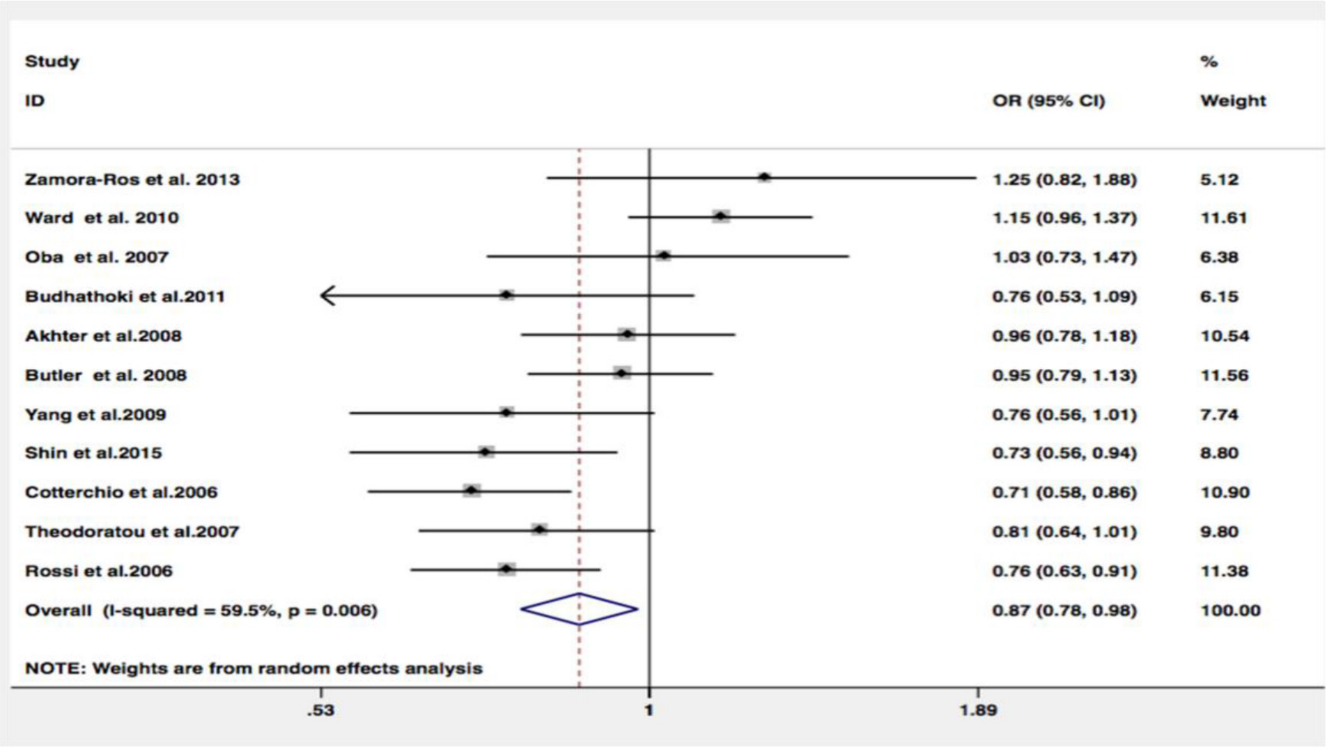

Supplementary Figure S7: Forest plot of isoflavones intake and colorectal cancer risk.

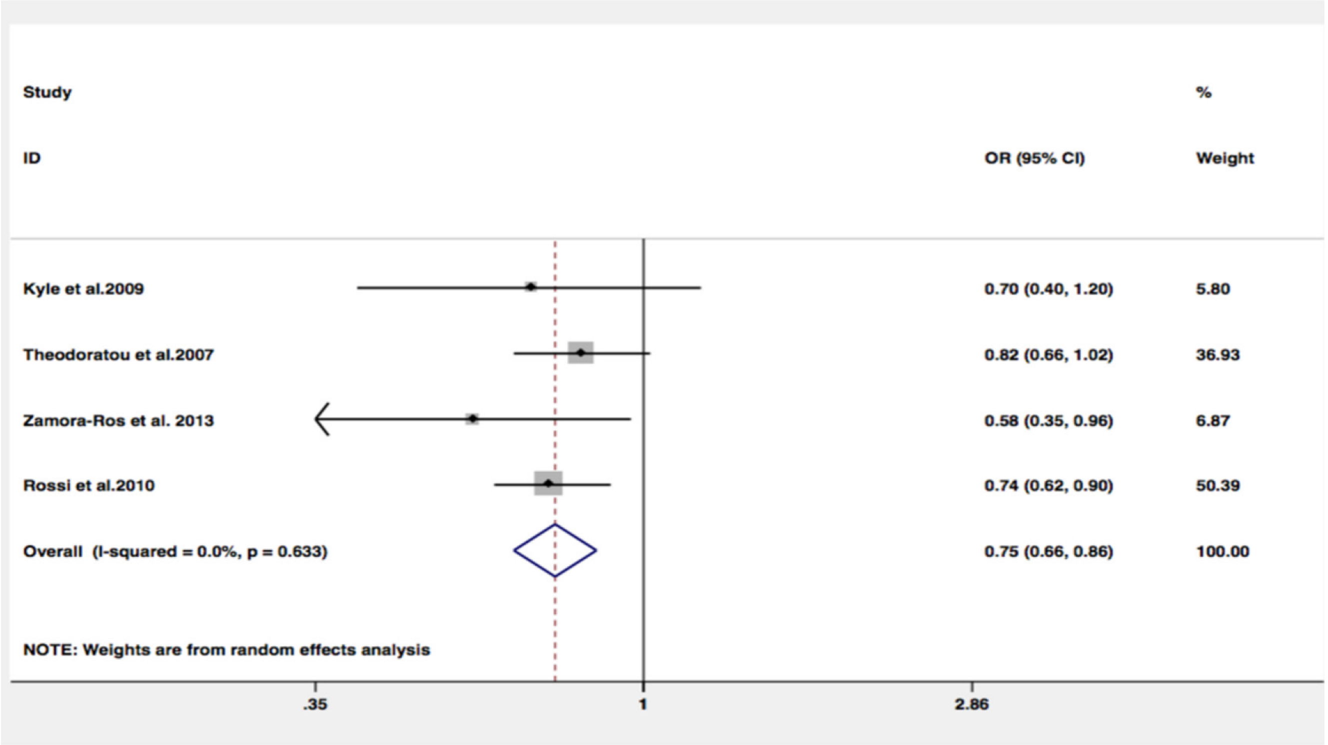

Supplementary Figure S8: Forest plot of procyanidins intake and colorectal cancer risk.
